# Current Management of Highly Calcified Coronary Lesions: An Overview of the Current Status

**DOI:** 10.3390/jcm12144844

**Published:** 2023-07-23

**Authors:** Gianluca Caiazzo, Carlo Di Mario, Elvin Kedhi, Giuseppe De Luca

**Affiliations:** 1 ICCU, San Giuseppe Moscati Hospital, ASL CE, 81031 Aversa, Italy; gianluca.caiazzo@gmail.com; 2 Structural Interventional Cardiology, Careggi University Hospital, 50134 Florence, Italy; carlo.dimario@unifi.it; 3 Erasmus Hospital, Université libre de Bruxelles (ULB), 1070 Brussels, Belgium; elvin.kedhi@erasme.ulb.ac.be; 4 Division of Cardiology, AOU Policlinico G Martino, 98124 Messina, Italy; 5 IRCCS Galeazzi-Sant’Ambrogio Hospital, 20157 Milan, Italy

**Keywords:** coronary calcified lesion, percutaneous coronary intervention, intracoronary

## Abstract

The amount of coronary calcium strongly correlates with the degree of atherosclerosis and, therefore, with the rate of future cardiac events. Calcified coronary lesions still represent a challenge for interventional cardiologists, bringing not only a higher risk of immediate complications during percutaneous coronary interventions (PCI), but also a higher risk of late stent failure due to under-expansion and/or malapposition, and therefore, have a relevant prognostic impact. Accurate identification of the calcified plaques together with the analysis of their distribution pattern within the vessel wall by intracoronary imaging is important to improve the successful treatment of these lesions. The aim of this review is to guide readers through the assessment of the calcified plaque distribution using intracoronary imaging in order to select the best devices and strategies for plaque debulking and lesion preparation.

## 1. Introduction

Coronary artery disease still represents the leading cause of mortality in developed countries [1,2]. Therefore, significant attention has been paid to the identification of new risk factors [3,4,5], improvement in pharmacological preventive strategies [6,7,8], and percutaneous treatment techniques [9,10,11,12,13]. However, the outcome remains unsatisfactory in special subsets of patients [14,15,16]. In particular, those with moderately and severely calcified lesions have been excluded from enrollment in most stent trials and still represent a challenge for interventional cardiologists, for many reasons. From a procedural point of view, it is well known that coronary calcium is an independent predictor of unsuccessful drug-eluting stent (DES) deployment and that its presence may damage the polymer/drug coating [17,18]. In terms of clinical outcomes, it has been widely demonstrated that the greater the arc, length, or thickness of calcium, the greater likelihood of stent under-expansion, which is associated with an increase in ischemic events including re-stenosis and stent thrombosis at 1 year [19,20]. Very recently, interesting long-term data showed that, at 10 years after PCI with new-generation DES, there was an increase in adverse events by the degree of coronary calcification and that the presence of heavily calcified lesions was an independent predictor of mortality, with a similar prognosis following PCI or CABG [21,22]. Advanced age, renal disease, and diabetes have all been associated with coronary artery calcification (CAC), with severe CAC affecting between 6 and 20% of patients treated with PCI [16]. This issue is expected to become more relevant in the next few years because of population aging and increased diabetes and chronic renal disease rates. Accurate identification of the calcified plaques together with the analysis of their distribution pattern within the vessel wall by intracoronary imaging is important to optimize the treatment of these lesions and might help in achieving better clinical outcomes [23]. Recognition of such calcified anatomy allows the appropriate use of ablative techniques for initial lesion modification and vessel preparation prior to DES implantation. Several tools and techniques have been proposed to overcome calcified lesions-related issues and some of them have shown significant efficacy and safety data. Buddy wires, guide extension catheters, and balloon anchoring are some of the possible options to cross calcified lesions. When successful treatment cannot be obtained using these options, a dedicated device (balloon-based or ablation-based) should be used since it is well recognized that treating stent under-expansion in a heavily calcified lesion is more difficult than preventing under-expansion.

## 2. Coronary Calcification in Human Atherosclerosis

Calcium in the coronary arteries has been used as a surrogate marker of coronary atherosclerosis since the 1940s [24]. The atherosclerotic process starts with the focal retention of apo B–containing lipoproteins within the subendothelial extracellular matrix [25,26]. This causes inflammation, resulting in the release of peptides, which attract monocytes that enter the tissue, become tissue macrophages, and phagocytize the lipoprotein cholesterol complex. In the process of catabolism, the lipoprotein cholesterol complex is aggregated and oxidized. Oxidized lipoprotein cholesterol toxicity for the macrophage can cause the death of the lipid-laden macrophage (foam cell) [27]. The inflammatory milieu stimulates angiogenesis, producing vessels with the typical fragility of unstable plaques. Ruptured intraplaque vessels result in intraplaque hemorrhages, increasing free cholesterol, and raising the likelihood of acute plaque rupture [28,29,30]. Interestingly, it has been proven that intimal and medial calcifications are different. Intimal calcification resembles endochondral bone formation in long bones and the progression of the lesion is likely driven by chondrocyte-like cells and associated with the expression of inflammatory factors, such as cytokines, whereas medial calcification has a different mechanism driven by the action of osteoblast-like cells [31]. Whether patients develop medial or intimal calcification is determined by local factors. Calcium distribution within the vessel wall is relevant in order to guide interventional cardiologists to select the best strategies for plaque modification and debulking.

## 3. Imaging Techniques for Calcified Plaques Identification

### 3.1. Coronary CT

Coronary CT angiography (CCTA) is the most important non-invasive imaging technique used to detect calcium. Calcium is detected as an area of hyper-attenuation of at least 1 mm^2^ with >130 Hounsfield units or ≥3 adjacent pixels using the Agatston method [32]. A coronary calcium score is calculated using a weighted value assigned to the highest density of calcification in each coronary segment (excluding calcium in the valves or aorta) that is then multiplied by the area and finally summed for all arteries to give a total coronary artery calcium score, which has been demonstrated to be a good prognostic tool for clinical events in the mid- to long-term in asymptomatic individuals [33,34]. On the basis of such evidence, CCTA is suggested for the identification of coronary atherosclerosis in symptomatic patients in the 2019 guidelines of the European Society of Cardiology for chronic coronary syndromes with a Class I recommendation, level of evidence B [35]. Of note, CCTA can detect spotty calcification, which is one of the four signs of vulnerable plaques (i.e., low CT attenuation, remarkable positive remodeling, spotty calcification, and the napkin-ring sign).

### 3.2. Coronary Angiography

**Figure 1:**
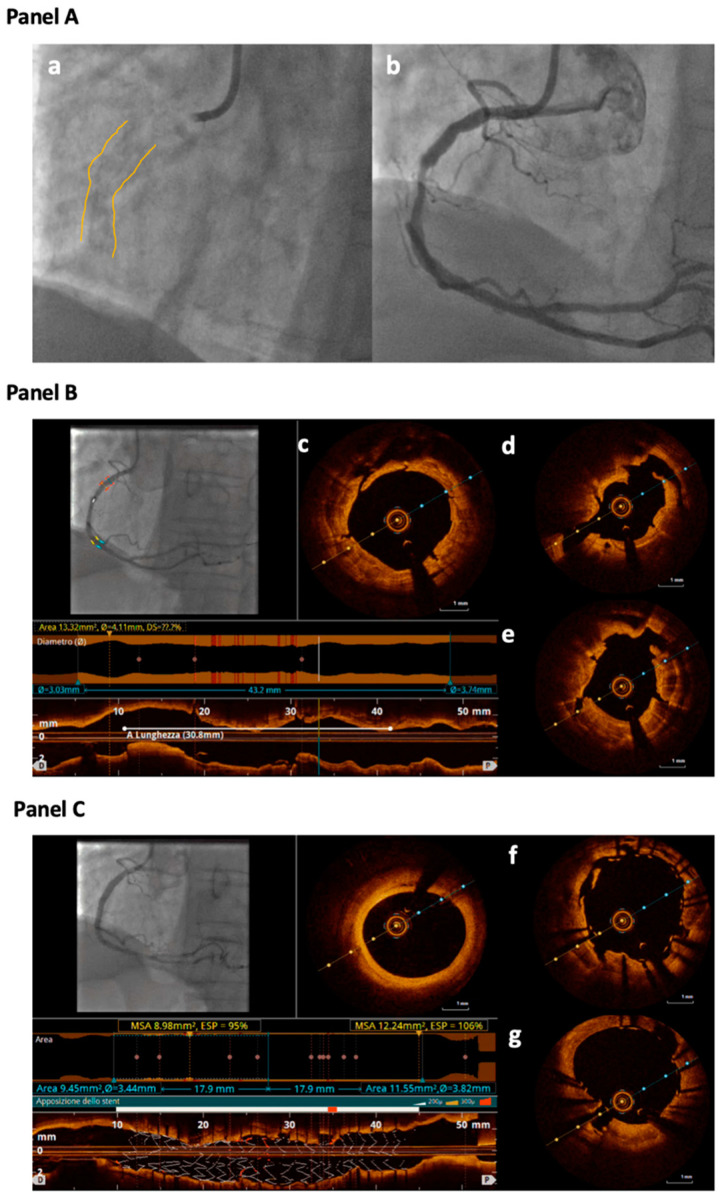
**Panel A**: Angiographic view of a calcified stenosis located in the mid-Right Coronary Artery (RCA) (**a**,**b**); in the still frame without contrast (**a**) severe calcification is identifiable as radiopacity visible on both sides of the arterial lumen, as a double track (highlighted by yellow contours). **Panel B**: Optical Coherence Tomography (OCT) evaluation of the RCA after treatment with 3.5 mm OPN balloon inflated at 30 atm. Different cross-sections show clear cracks into the calcific concentric plaque; small cuts not penetrating through the entire plaque are identifiable (**c**) as well as bigger cracks cutting the entire calcific plaque (**d**,**e**). **Panel C**: OCT evaluation of the RCA after stent deployment showing good struts apposition with cracks still evident behind the stent struts (**f**,**g**).

The amount of coronary calcium is often not well identified by coronary angiography and its depth within the plaque is not assessed. Severe calcification is defined as radiopacity observed without cardiac motion, as a double track visible on both sides of the arterial lumen (Figure 1, panel a). In a study by Mintz and colleagues, the sensitivity of angiography in detecting the presence of target lesion calcium was 48% when compared to IVUS, and it was the lowest in those lesions with one-quadrant calcium and the highest (85%) in lesions with four-quadrant calcium; the overall specificity of the angiographic detection of target lesion calcium was 89% [36]. The lower capacity showed by coronary angiography in identifying calcified plaques was recently confirmed in a study by Wang et al., where calcium was detected by angiography in only 40.2% of lesions [37]. In that study, IVUS detected any amount of calcium in 82.7% of lesions and OCT in 76.8% of lesions. Interestingly, any disagreement between coronary angiography and IVUS/OCT was due to thin calcium deposits that were demonstrated not to inhibit stent expansion. For this reason, despite the intrinsic lower diagnostic performance showed by angiography, angiographically visible calcium (thick calcium) seemed to be a good marker to predict stent under-expansion.

### 3.3. Intravascular Ultrasound (IVUS)

**Figure 2:**
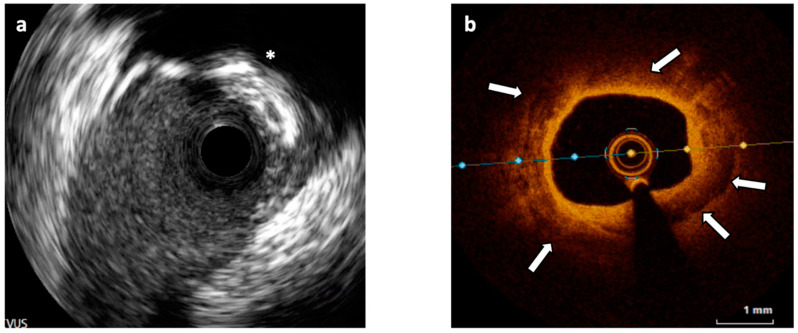
(**a**) Intravascular Ultrasound (IVUS) cross-section showing a 180° calcified plaque opposite to a side branch origin and producing shadow (asterisk). (**b**) Optical Coherence Tomography (OCT) cross-section of a different segment of the vessel showing an almost concentric calcified plaque visible as signal-poor regions with sharply delineated borders (white arrows).

Intravascular ultrasound (IVUS) is the first catheter-based technology used for intravascular imaging, introduced by Yock et al. in the 1980s [38]. This technology offers a relatively low resolution when compared to other imaging tools (i.e., OCT) but higher penetration depth. Its axial resolution is 100–150 μm and lateral resolution is 150–300 μm for 40 MHz, whereas, for 60 MHz, it ranges between 40–60 μm and 60–140 μm for axial and lateral resolutions, respectively [39]. Such resolution characteristics are not optimal when it is the case to assess superficial plaques or suboptimal PCI results. At IVUS analysis, calcium is hyperechoic, brighter than the reference adventitia, with shadowing (Figure 2, panel a); for this reason, the thickness of calcium cannot be detected. Such a characteristic pattern might be partly shared by fibrous plaques as well, but calcium produces distinctive reverberations at reproducible distances, especially after treatment with ablative techniques. Semi-quantitative analysis is possible by assessing calcium arc and calcium length [38,39]. From a qualitative point of view, calcium can be described as superficial (acoustic shadowing located within the most superficial 50% of the plaque and media thickness) or deep (acoustic shadowing within the deepest 50% of the plaque and media thickness) [19]. Of interest, an IVUS-based calcium score has been used to identify calcified stenoses at risk of stent under-expansion and requiring adjunctive calcium modification before stent implantation. Plaque characteristics included in the score are: a superficial calcium angle > 270° longer than 5 mm, 360° of superficial calcium, a calcified nodule, and a vessel diameter < 3.5 mm [40]. Given the limitations of the qualitative visual interpretation of grey-scale IVUS images, several post-processing methods have been developed to optimize coronary plaque tissue characterization, such as VH-IVUS (virtual histology), iMAP-IVUS (iMap-Intravascular Ultrasound Radiofrequency Signal Analysis), or IB-IVUS (integrated backscatter) [41,42]. The identification of calcified nodules represents an important step during the morphologic evaluation of the plaque, since their treatment is difficult and related to procedural complications. IVUS characteristics of a calcified nodule are a convex shape on the luminal surface, a convex shape on the luminal side of calcium, and an irregular luminal surface [19]. The identification of intra-plaque microcalcifications is rarely possible using IVUS and this represents a major limitation, since it has been suggested that microcalcifications (>5 mm) in fibrous caps of fibroatheromas can increase local tissue stress and promote cavitation-induced plaque rupture [19].

### 3.4. Optical Coherence Tomography (OCT)

OCT uses near-infrared light directed at the vessel wall through a rotating single optical fiber coupled with an imaging lens within a short-monorail imaging sheath. By measuring the amplitude and time delay of the backscattered light, OCT generates high-resolution, cross-sectional, and three-dimensional volumetric images of the vessel microstructure [43].

The shorter wavelength of the infrared light in OCT (1.3 μm) compared with ultrasound in IVUS (~40 μm at 40 MHz) allows greater axial resolution (10–20 μm versus 50–150 μm) but lower penetration depth (1–2 mm versus 5–6 mm), which limits OCT imaging, particularly in the presence of highly attenuating structures such as red thrombus or lipid/necrotic core.

As mentioned, in calcified lesions, IVUS delineates the calcification arc but not its thickness because of the reflection of ultrasound waves off calcium; in contrast, OCT allows the determination of both the calcification arc and thickness in most cases, since it identifies calcified plaques as signal-poor regions with sharply delineated borders (Figure 2, panel b). As for IVUS, an OCT-based scoring system has been validated in calcified lesions to help determine which calcific morphologies lead to stent under-expansion: calcium arc > 180° (2 points), calcium length > 5 mm (1 point), and calcium thickness > 0.5 mm (1 point) were associated with poor stent expansion [44]. Therefore, an OCT-based calcium score of ≥3 may indicate the need for calcium modification to induce calcium fracture, which is associated with enhanced stent expansion [45]. In the study by Wang et al., the sensitivity and specificity of angiography to detect any OCT calcium were 50.9% and 95.1%, and the sensitivity and specificity of angiography to detect any IVUS calcium were 48.4% and 98.7%, respectively [46]. It has been widely demonstrated that intravascular imaging-guided PCIs in calcified lesions achieve better procedural and clinical outcomes when compared to angiography-guided procedures [23,46]. An interesting step forward in the field of coronary calcification investigation and treatment is represented by the recent iteration of the OCT technology (Ultreon™ 1.0 Software; Abbott, Chicago, IL, USA) powered by artificial intelligence and enables the automatic quantification of calcification and vessel sizing.

## 4. Imaging-Guided Plaque Modification

**Figure 3:**
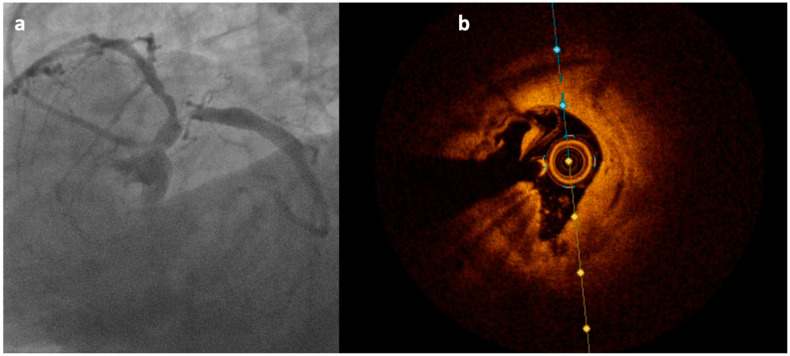
Calcific plaque located at the level of the ostial left circumflex coronary artery causing evident sub-occlusive stenosis at angiography (**a**); OCT analysis (performed after 2.5 mm balloon pre-dilation) clarifies the nodular origin of the plaque not detectable at angiography (**b**).

**Figure 4:**
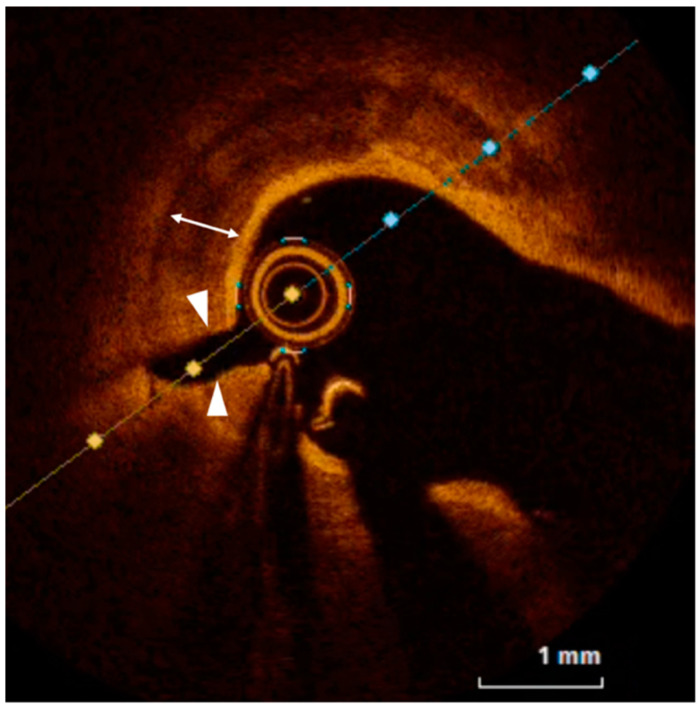
OCT evaluation of a thick (white arrow) calcified plaque after treatment with cutting balloon showing a clear full-thickness crack (arrow heads) in the calcified plaque.

**Figure 5:**
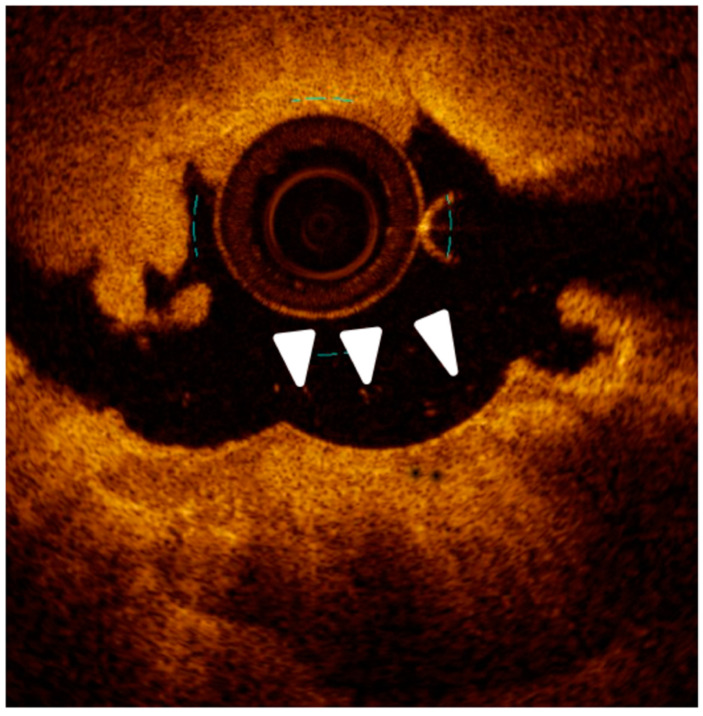
Optical Coherence Tomography (OCT) cross-section showing a thick calcific plaque (lower quadrants) treated with rotational atherectomy.

The importance of the calcified plaque pattern assessment in intracoronary imaging analysis lies in the possibility of selecting different treatment strategies. As a matter of fact, such selection has to take the capacity of the different devices to cross the lesion into account. Based on the above considerations, De Maria et al. proposed a comprehensive algorithm indicating balloon-based techniques (non-compliant, scoring/cutting, high-pressure, lithotripsy) in case of crossable lesions and ablative techniques (rotational atherectomy, orbital atherectomy, excimer laser) for non-crossable lesions [47]. According to the calcium location, it can be determined that when calcium is superficial, successful treatment depends on the thickness, length, and arc of calcium: if the calcium thickness is greater than 0.5 mm, the arc is >180°, and the length is greater than 5 mm, balloon-based strategies are not usually effective and ablative techniques are recommended; the same approach should be considered when facing calcified nodules (Figure 3). On the other hand, when calcium is located deep in the arterial wall and covered by superficial fibrosis adjacent to the lumen, it can reasonably be approached with conventional non-compliant high-pressure balloons, cutting/scoring balloons, and/or lithotripsy (Figure 4) [45,46,47]. Whether one calcified plaque characteristic is more impactful on stent under-expansion among others is unknown to date. Based on the existing scores, all the plaque characteristics included in the IVUS-based score seem to have the same relevance (one point each), whereas, in the OCT-based score by Fujino et al. [46], the calcium arc > 180° has been found to weigh more (two points). Of note, the presence of a thinner calcific plaque (<0.67 mm) and a >227 degrees of concentric calcific distribution have been found to be associated with higher chances of cracking the calcium and of optimal stent expansion [48]. Furthermore, some technical tricks relative to the use of imaging tools can be extrapolated to acquire useful information: as an example, the position of the imaging catheter in relation to the plaque might be used to anticipate the possible impact of the use of the Rotablator burr in that area. If the imaging catheter is adherent to the side of the vessel wall, away from the calcific component, then a larger burr should be selected (Figure 5) [49].

## 5. Balloon-Based Techniques

### 5.1. Non-Compliant Balloons/OPN

The main characteristic of non-compliant balloons (NC) resides in the possibility to be inflated at high pressures with no significant increase in diameter, allowing the application of higher forces in a focal segment of a coronary vessel with less rates of coronary dissections or perforations due to the “dog-bone” effect [50]. The OPN NC balloon (SIS Medical, Frauenfeld, Switzerland) represents a “super NC-balloon” with minimal increases in diameter when very high pressures are applied and such a feature is subtended by a twin-layer technology. This balloon has a rated burst pressure of 35 atmospheres, but the balloon was tested up to 45 atmospheres (Figure 1, panel b). The main disadvantage of this device is that, due to its high profile (0.028 inches), it has the poor capability to cross the stenotic lesions when compared to other dedicated devices [39]. Its performance has been recently tested in several studies [51,52,53]. Secco et al. [51] included a consecutive series of 91 lesions where NC balloons at high pressures failed to achieve an adequate post-dilatation luminal gain and were, therefore, treated with an OPN NC balloon up to 40 atm. Angiographic success was obtained in 84 lesions (92.3%). All of the remaining lesions received rotational atherectomy. MLD and acute gain were significantly greater and %DS was significantly lower post OPN NC balloon compared with conventional NC balloon inflation (*p* < 0.001). No coronary perforations occurred. No acute or 30-day follow-up MACE was reported. The randomized ISAR-CALC trial showed similar good results in terms of lesion preparation when compared to scoring balloons, and a better, although not statistically significant, angiographic result [52]. A recent compelling retrospective registry focused on patients with calcified lesions and treated with OCT-guided OPN-based PCI showed a stent expansion ≥ 80% in 80% of cases with a mean final expansion post intervention of 85.7% ± 8.9; of interest, no perforations and no-reflow occurred [53].

### 5.2. Scoring and Cutting Balloons

**Figure 6:**
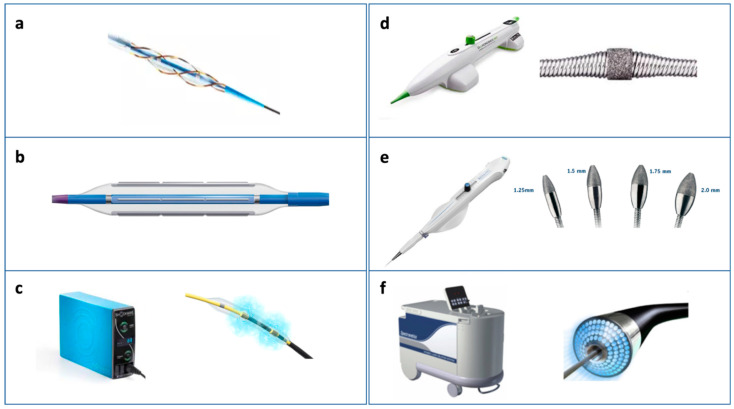
Balloon-based techniques (**a**–**c**) for crossable lesions and ablative techniques (**d**–**f**) for non-crossable lesions.

Scoring balloons are semi-compliant balloons with scoring elements located on the surface, which allow focal concentration of the force during inflation and increase balloon stability during inflations (Figure 6, panel a). The latter characteristic is especially useful when treating typical fibrous plaques of re-stenotic stents caused by neo-intimal hyperplasia and is also shared by cutting balloons [54,55]. The peculiar structural feature characterizing cutting balloons is represented by three or four metal micro-blades longitudinally placed on the surface of the balloon and cutting the media with radial incisions when the balloon is inflated; this allows the reduction in the elastic recoil and counteracts neointima proliferation (Figure 4 and Figure 6, panel b). Caution should be paid when deciding to re-cross a cutting balloon through the struts of a previously implanted metallic stent because of the risk of entanglement. When compared to traditional plain-only balloon angioplasty (POBA) in the past, cutting balloons have shown no difference in terms of six-month binary restenosis but a higher rate of perforation. However, recent data relative to newer iterations of such devices have shown the same acute cross-sectional area gain obtained when compared to scoring balloons but better performance in lesion crossing [56].

### 5.3. Intravasular Litotripsy (IVL)

Intravascular lithotripsy represents a recently introduced strategy (CE mark in May 2017) for the treatment of calcified coronary lesions based on the principles of lithotripsy that has been used to break up stones in the kidneys for over 30 years (Figure 6, panel c). The Shockwave Medical (Santa Clara, California) IVL system consists of a 0.014-inch guidewire-compatible, fluid-filled balloon angioplasty catheter with two lithotripsy emitters incorporated into the shaft (the distal emitter is slightly more central to enhance flexibility, whereas the proximal emitter is located near the proximal end of the balloon) [57,58]. The last iteration of the catheter can provide up to 120 total IVL pulses and is intended for single use. The emitters convert electrical energy into transient acoustic pressure pulses that impact calcium with expanding and collapsing vapor bubbles, creating a short burst of acoustic pressure waves. These pressure waves travel through the vessel tissue with an effective pressure of 50 atmospheres and create both deep and superficial calcium fractures. The effect on deep calcium is a major benefit of lithoplasty compared with other ablation techniques. IVL represents a user-friendly technique with a short learning curve and these are some of the reasons that explain why it is becoming a standard approach when facing calcified coronary lesions. One more advantage over ablative techniques is that it is ideal for bifurcation lesions, including left main coronary disease, as the operator can wire and protect both major branches during lesion preparation with no significant downstream debris released [58,59]. IVL has been initially evaluated in small single-arm, non-randomized studies, which have demonstrated high rates of device success with excellent early angiographic as well as late clinical outcomes [60,61]. The DISRUPT CAD III trial was a prospective, single-arm multicenter study designed for regulatory approval of coronary IVL, where IVL was demonstrated to be safe and effective with a low rate of major complications. In particular, in that study, MACE and target lesion failure (TLF) over 30 days occurred in 7.8% and 7.6% of patients and was primarily driven by target vessel MI. There were two deaths (0.5%) within 30 days. Angiographic complications were one severe dissection (Type D–F) and one perforation (0.3%). At OCT examination, multiplane and longitudinal calcium fractures after IVL in 67.4% of lesions were demonstrated, with excellent stent expansion in those with and without calcium fractures identified by OCT [62].

## 6. Calcium-Ablation Techniques

### 6.1. Rotational Atherectomy

Intracoronary imaging studies have clearly shown that rotational atherectomy ablate calcium causes fissuring or cracks within the ablated calcium and, as mentioned, its use is suggested when superficial luminal calcium is found at imaging analysis and/or when non-crossable stenoses are to be treated [63,64,65]. The described differential cutting operated by rotational atherectomy is supposed to allow the mechanical ablation of hard fibrocalcific plaques while sparing adjacent elastic tissue that deflects away from the ablating burr (Figure 5). The Rotablator System (Boston Scientific) is made up of a nickel-plated elliptic burr coated with diamond microscopic crystals, a single advancer that can transmit rotational speed to the burr, and is connected with a gas-driven turbine and a control console and foot pedal or an activator in the connecting handle (Figure 6, panel e) [39,66]. The most recent indications relative to the use of a Rotablator recommend a smaller burr size and standardized protocols (i.e., rotation speeds between 135,000 and 180,000 rpm) in order to reduce procedural complications [67]. Dedicated 330-mm long wires are available but their performance in heavily calcified vessels is not always optimal so they are often inserted through an over-the-wire balloon or microcatheter after a work-horse standard coronary wire has been used. Of note, adjunctive wires are not allowed during rotablation to avoid wire cutting or perforation. From a practical point of view, short burr runs are usually preferred and fluoroscopic, acoustic, and tactile signals should be monitored to avoid significant deceleration in rotational speed (>5000 rpm), which is associated with complications [66,67]. The latter is also achieved thanks to the “pecking motion” technique, a forward-backward movement of the burr, ideated to reduce the effective ablation time. Although rotational atherectomy is still considered one of the best tools for debulking in calcified lesions, it has been found that in European countries the rate of rotational atherectomy as a function of the total PCI number is still low (0.8–3.1%) [67]. Such evidence is partly explained by the concern regarding the complexity of the Rotablator procedures and potential procedure-related complications occurring in the absence of standardized protocols. Conflicting evidence has been shown in the past, linked to the use of rotational atherectomy before stenting. The Rotational Atherectomy Prior to Taxus Stent Treatment for Complex Native Coronary Artery Disease (ROTAXUS) trial found a 9-month higher late lumen loss in the rotablation group compared to the group of stented patients without the use of rotablation; in-stent binary restenosis, target lesion revascularization, definite stent thrombosis, and major adverse cardiac events were similar in both groups [68]. Interesting findings were recently made by pooling patient-level data from the PREPARE-CALC (Comparison of Strategies to Prepare Severely Calcified Coronary Lesions) and ISAR-CALC (Comparison of Strategies to Prepare Severely Calcified Coronary Lesions) randomized trials. In this study, Rheude et al. sought to compare rotational atherectomy versus balloon-based techniques before drug-eluting stent implantation in severely calcified coronary lesions. Two-hundred patients with available OCT data were included and lesion preparation was obtained with rotablation; a modified balloon and a super high-pressure balloon were compared [69]. Of note, strategy success was more frequent with rotablation versus modified balloons and super high-pressure balloons, but clinical outcomes did not differ among groups. Rotational atherectomy has also recently been proven to be safe and effective for the treatment of calcific left main artery stem lesions at one-year follow-up, yielding comparable outcomes to rotablation-based PCIs performed on non-left main lesions [70].

### 6.2. Orbital Atherectomy

Orbital Atherectomy uses a different mechanism to reduce the calcified plaque burden while minimizing the damage to the non-calcified tissue. It is based on the Diamondback 360° Coronary Orbital Atherectomy System (OAS) (Cardiovascular Systems Inc., St. Paul, MN, USA), a percutaneous system that takes advantage of centrifugal force to modify calcified lesions. It has an eccentrically mounted diamond-coated crown that orbits over an atherectomy guide wire at high speeds (Figure 6, panel d). The position of the crown within the vessel is controlled via a control handle. The crown’s orbital diameter radially expands via centrifugal force. The OAS promises several advantages over the Rotablator: first, the average particle size created by OAS is much smaller than that produced by rotational atherectomy and can be removed through the reticuloendothelial system. Second, by increasing its orbit as rotational speed increases, Orbital Atherectomy allows for the ablation of calcium using the same device (1.25-mm crown) in larger vessels (up to 3.5 mm in diameter). Third, a bidirectional atherectomy can be performed, not only in anterograde as in rotational atherectomy, with a consequent decrease in crown entrapment. Fourth, the continuous flow of blood and saline solution or other lubricant solutions during ablation reduces thermal injury, potentially decreasing no-reflow and periprocedural complications [39,49,71]. The safety and efficacy of this system have been widely tested in the ORBIT trials. The 3-year results of the ORBIT II trial, a single-arm trial including 443 patients with de novo severely calcified coronary lesions treated with OAS, have shown a rate of MACE as low as 23.5%, including cardiac death, MI, and TVR. The 3-year target lesion revascularization rate was 7.8% [71].

### 6.3. Excimer Laser

IVUS and OCT studies have shown that Excimer Laser coronary angioplasty (ELCA) is not able to decrease lesion-associated calcium because the ablative effects on calcium are minimal and success relies on ablation of the softer tissues within the calcific lesion. This causes dissections and fragmentation of calcific deposits, presumably as part of the photoacoustic effect [19]. Since the mid-1990s, lasers have been used in coronary and peripheral procedures, mainly at a low to medium energy level, regardless of the lesion to be treated. This caused initial disappointing results, with low procedural success rates, especially in the case of complex calcified lesions. The introduction of strategies using higher energies with dedicated techniques (contrast medium, type of probe, anterograde and/or retrograde passages, number of laser passages) significantly changed the effectiveness of this tool [72,73,74]. The system consists of an excimer laser generator, the CVX-300 unit (Philips), and a series of pulsed xenon-chlorine laser catheters capable of delivering excimer energy via optical fibers (Figure 6, panel f). Tissue ablation is mediated by three distinct mechanisms: photochemical, photothermal, and photomechanical. The products of photoablation are <10 μm in diameter and are easily filtered by the reticuloendothelial system with trivial consequences for the microvascular bed [75]. The efficacy of this technique in calcified lesions has been improved by the use of contrast injection at the highest fluence and repetition rate (i.e., 80 mJ/mm^2^ and 80 Hz for the 0.9 mm catheter), the so-called “explosion technique”, and represents a good bail-out strategy for unexpanded stents [76].

## 7. Conclusions

With the population aging, the presence of coronary calcified lesions is meant to increase. Several effective tools and techniques have been developed to address this issue. The use of intracoronary imaging represents an important procedural step to accurately analyze plaque composition and distribution. This allows the selection of the most appropriate strategy and device in order to treat such lesions and achieve better procedural and clinical outcomes.

## Data Availability

Not applicable.
